# Epistatic Relationships between *sarA* and *agr* in *Staphylococcus aureus* Biofilm Formation

**DOI:** 10.1371/journal.pone.0010790

**Published:** 2010-05-24

**Authors:** Karen E. Beenken, Lara N. Mrak, Linda M. Griffin, Agnieszka K. Zielinska, Lindsey N. Shaw, Kelly C. Rice, Alexander R. Horswill, Kenneth W. Bayles, Mark S. Smeltzer

**Affiliations:** 1 Department of Microbiology and Immunology, University of Arkansas for Medical Sciences, Little Rock, Arkansas, United States of America; 2 Department of Biology, University of South Florida, Tampa, Florida, United States of America; 3 Department of Microbiology and Cell Science, University of Florida, Gainesville, Florida, United States of America; 4 Department of Microbiology, University of Iowa, Iowa City, Iowa, United States of America; 5 Department of Pathology and Microbiology, University of Nebraska Medical Center, Omaha, Nebraska, United States of America; University of Liverpool, United Kingdom

## Abstract

**Background:**

The accessory gene regulator (*agr*) and staphylococcal accessory regulator (*sarA*) play opposing roles in *Staphylococcus aureus* biofilm formation. There is mounting evidence to suggest that these opposing roles are therapeutically relevant in that mutation of *agr* results in increased biofilm formation and decreased antibiotic susceptibility while mutation of *sarA* has the opposite effect. To the extent that induction of *agr* or inhibition of *sarA* could potentially be used to limit biofilm formation, this makes it important to understand the epistatic relationships between these two loci.

**Methodology/Principal Findings:**

We generated isogenic *sarA* and *agr* mutants in clinical isolates of *S. aureus* and assessed the relative impact on biofilm formation. Mutation of *agr* resulted in an increased capacity to form a biofilm in the 8325-4 laboratory strain RN6390 but had little impact in clinical isolates *S. aureus*. In contrast, mutation of *sarA* resulted in a reduced capacity to form a biofilm in all clinical isolates irrespective of the functional status of *agr*. This suggests that the regulatory role of *sarA* in biofilm formation is independent of the interaction between *sarA* and *agr* and that *sarA* is epistatic to *agr* in this context. This was confirmed by demonstrating that restoration of *sarA* function restored the ability to form a biofilm even in the corresponding *agr* mutants. Mutation of *sarA* in clinical isolates also resulted in increased production of extracellular proteases and extracellular nucleases, both of which contributed to the biofilm-deficient phenotype of *sarA* mutants. However, studies comparing different strains with and without proteases inhibitors and/or mutation of the nuclease genes demonstrated that the *agr*-independent, *sarA*-mediated repression of extracellular proteases plays a primary role in this regard.

**Conclusions and Significance:**

The results we report suggest that inhibitors of *sarA*-mediated regulation could be used to limit biofilm formation in *S. aureus* and that the efficacy of such inhibitors would not be limited by spontaneous mutation of *agr* in the human host.

## Introduction

Biofilm formation is an important aspect of many *Staphylococcus aureus* infections including endocarditis, osteomyelitis, and infections of implanted medical devices. This is true not only with respect to the pathogenesis of the infection itself but also with respect to antimicrobial therapy. Indeed, the presence of a biofilm limits the efficacy of antimicrobial therapy to the point that surgical intervention is often required to remove infected tissues and/or implanted devices [1]. For this reason, a considerable research effort has been aimed at defining the mechanistic basis of *S. aureus* biofilm formation. These studies have focused on the role of both individual components and the regulatory factors that modulate the production of these components. To date, over 20 genes have been implicated, with approximately half of these serving a regulatory role [2]. We chose to focus on the accessory gene regulator (*agr*) and the staphylococcal accessory regulator (*sarA*) because both of these loci have been shown to play central roles in *S. aureus* regulatory circuits that includes important but generally opposing roles in biofilm formation. Specifically, while there is one report to the contrary [3], most studies have concluded that expression of *agr* limits biofilm formation [4]–[6], and it has been suggested that this may serve as a means for the dispersal of *S. aureus* from an established biofilm [7]–[9]. In contrast, expression of *sarA* has consistently been shown to promote biofilm formation in both *S. aureus* and *S. epidermidis* [4]–[6], [10], [11].

There are also reports demonstrating that the opposing roles of *sarA* and *agr* in biofilm formation are therapeutically relevant. For instance, we demonstrated that mutation of *sarA* can be correlated with increased susceptibility to the functionally-diverse antibiotics daptomycin, linezolid and vancomycin in the specific context of an established biofilm [12], [13]. At least under *in vitro* conditions, this increased susceptibility was evident even after taking into account the reduced capacity of a *sarA* mutant to form a biofilm [12]. In contrast, induction of *agr* expression was shown to result in the detachment of *S. aureus* cells from an established biofilm resulting in increased susceptibility to diverse antibiotics including rifampicin and levofloxacin [7], [8], [14]. Other reports have demonstrated that *agr* mutants accumulate within a biofilm and ultimately become the predominant subpopulation [6]. There is also a report demonstrating that the loss of *agr* function may confer a selective advantage *in vivo*, particularly under the pressure of vancomycin therapy [15]. Taken together, these results suggest that the opposing roles of *sarA* and *agr* in biofilm formation can be directly correlated with antibiotic susceptibility, with expression of the first leading to biofilm-associated intrinsic resistance and expression of the second having the opposite effect. This makes it important to define the epistatic relationships between *sarA* and *agr* in the context of biofilm formation.

Because both *sarA* and *agr* play global regulatory roles in *S. aureus* [16]–[18], it is not obvious why either would have an impact on biofilm formation. Moreover, expression of *sarA* is generally associated with increased expression of *agr* [16], [19]–[21], and based on this it might be anticipated that these two loci would play consistent rather than opposing roles with respect to each other. However, *sarA* also modulates expression of many genes independently of *agr*, examples of which include the genes encoding extracellular proteases and nucleases [22], [23]. Specifically, mutation of *agr* results in reduced production of these enzymes while mutation of *sarA* has the opposite effect [17], [23], [24]. Additionally, extracellular DNA (eDNA) has been shown to contribute to biofilm formation in *S. aureus* [25], and limiting nuclease or protease production by mutagenesis or the use of inhibitors of protease activity has been shown to promote biofilm formation and to partially restore biofilm formation in a *sarA* mutant [8], [14], [23], [26].

Collectively, these studies suggest that the opposing roles of *agr* and *sarA* in biofilm formation are due to the fact that the first induces while the second represses the production of extracellular proteases and/or nucleases. This is consistent with our results comparing the clinical isolate UAMS-1 with the commonly-studied 8325-4 laboratory strain RN6390. Specifically, by comparison to RN6390, UAMS-1 expresses *agr* at much lower levels, produces reduced amounts of extracellular proteases, and forms a more robust biofilm [10], [24], [27]. Additionally, mutation of *agr* enhances biofilm formation in RN6390 but has little impact in UAMS-1 and, conversely, mutation of *sarA* results in increased protease production and decreased biofilm formation in UAMS-1 but has little impact in RN6390 [10]. All *S. aureus* strains derived from 8325, including the 8325-4 strain RN6390, have genetic defects that have been shown to contribute to its high level of *agr* expression [28]–[30], and restoration of the *rsbU* defect in RN6390 has been correlated with decreased expression of *agr*, decreased production of extracellular proteases, and an increased capacity to form a biofilm [8]–[29]. However, community-acquired, methicillin-resistant *S. aureus* (CA-MRSA) isolates of the USA300 clonal lineage, which are increasingly prominent worldwide [31], are closely related to 8325-derived strains and also express *agr* at high levels [17], [32], [33]. As a result, they produce extracellular toxins at high levels [34], and this contributes to their ability to cause invasive disease even in otherwise healthy individuals [35], [36].

The production of extracellular proteases is also under the regulatory control of *agr*, and this suggests that the increased toxin production in USA300 isolates would be correlated with increased protease production and may therefore come at the expense of the ability to form a biofilm. Support for this hypothesis comes from a recent report demonstrating that mutation of *agr*, the inclusion of inhibitors of extracellular protease activity, and the mutation of specific genes encoding extracellular proteases can all be correlated with an enhanced capacity to form a biofilm in the USA300 isolate LAC [7], [8], [14]. However, to date, this issue has not been examined in a comprehensive manner, and it remains unclear whether expression of *agr* has a significant impact on biofilm formation in USA300 isolates in general and, if so, whether this limits the regulatory role of *sarA* in this respect. Based on this, we examined the impact of *sarA* and *agr* on biofilm formation in diverse clinical isolates with a specific emphasis on CA-MRSA isolates of the USA300 clonal lineage.

## Results and Discussion

### The impact of *agr* on biofilm formation

We previously demonstrated that clinical isolates of *S. aureus* generally form a more robust biofilm than the 8325-4 laboratory strain RN6390 and that biofilm formation in the latter is enhanced by mutation of *agr* [10]. This suggests that the level of *agr* expression in RN6390 can be functionally defined as excessive at least in the context of biofilm formation. The fact that clinical isolates of the USA300 clonal lineage are genotypically related to isolates of the 8325 lineage [32] and also express *agr* at high levels [8] led us to question whether they might also have a reduced capacity to form a biofilm and, if so, whether this could also be overcome by mutation of *agr*. To examine this issue, we chose three USA300 isolates, the genomes of two of which (FPR3757 and TCH1516, designated here as UAMS-1782 and UAMS-1790) have been sequenced [32], [37] while the third (UAMS-1625) was isolated from a patient with a fatal brain abscess [38].

**Figure 1:**
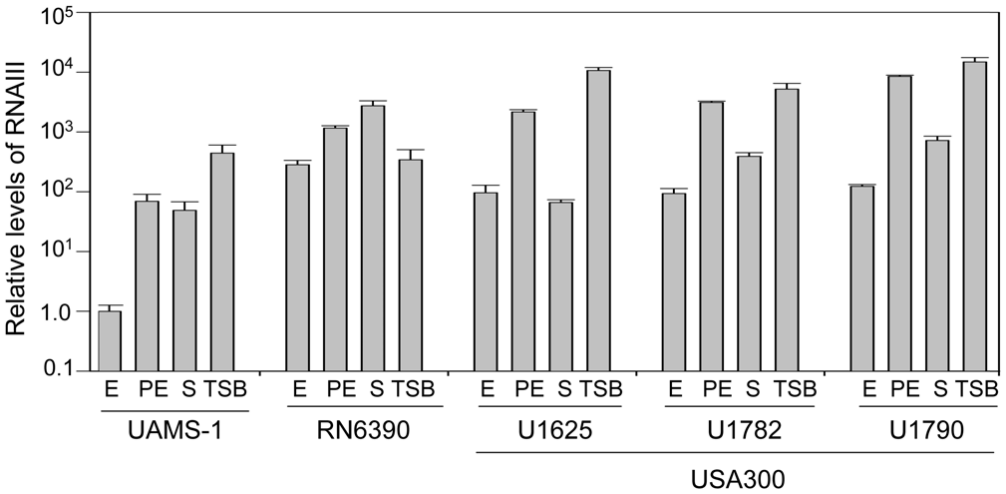
Production of RNAIII as a function of strain, growth phase, and growth medium. RNA was extracted from each strain grown in biofilm medium (BM) during the exponential (E, OD_560_ = 1.0), post-exponential growth phase (PE, OD_560_ = 3.0) and stationary (S) phases and the amount of RNAIII determined by qRT-PCR. RNA was also isolated from stationary-phase cultures grown in TSB. The value observed with UAMS-1 during the exponential growth phase was set at 1.0 with the results observed for other strains shown relative to this value. Results are shown as the mean ± the standard deviation of triplicate samples. Statistical analysis of the results observed in stationary-phase samples grown in BM confirmed a significant difference between RN6390 and all other strains and between UAMS-1782 and UAMS-1790 by comparison to both UAMS-1 and UAMS-1625.

The levels of RNAIII produced by each strain were dependent on both growth phase and growth medium. RNAIII levels were higher in TSB than in biofilm medium (BM) in all strains except RN6390, which produced comparable levels of RNAIII whether grown in BM or TSB (Fig. 1). The reduced production of RNAIII in BM is consistent with reports demonstrating that RNAIII production is repressed in medium supplemented with glucose [7], [24]. Nevertheless, all clinical isolates produced RNAIII in the expected growth-phase dependent pattern when grown in BM in that, by comparison to exponential growth, the production of RNAIII was increased in all strains in the post-exponential growth phase while, by comparison to the post-exponential growth phase, RNAIII levels were decreased in all strains in stationary-phase cultures (Fig. 1). RN6390 was once again an exception in that the levels of RNAIII observed in this strain were generally consistent across all growth phases.

**Figure 2:**
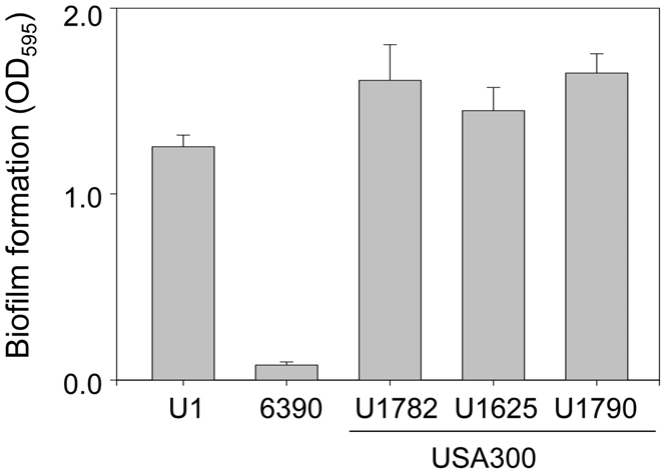
Biofilm formation in isolates of the USA300 clonal lineage. Biofilm formation was assessed using a microtiter plate assay as previously described [10]. Results are shown as the mean ± the standard deviation of 6 replicate samples from each strain. Statistical analysis confirmed a significant difference between RN6390 and each of the other strains.

Of the planktonic growth conditions examined in this report, the most applicable by comparison to our biofilm assays is a stationary-phase culture in biofilm medium, and under these conditions the targeted strains could be divided into three groups. The first consisted solely of RN6390, which produced higher levels of RNAIII by comparison to all other strains. The second consisted of the USA300 isolates UAMS-1782 and UAMS-1790, both of which produced RNAIII at levels that exceeded those observed in UAMS-1 or the USA300 isolate UAMS-1625 to a statistically-significant degree (Fig. 1). These results demonstrate that, while USA300 isolates generally produced RNAIII at levels that exceeded those observed in UAMS-1, they were nevertheless below those observed in RN6390. More importantly, this difference appears to be biologically relevant in that, as defined by our microtiter plate assay, all USA300 isolates formed a biofilm that was comparable to that observed with UAMS-1 and significantly greater than that observed with RN6390 (Fig. 2).

The fact that all three USA300 isolates formed a biofilm in our assay is in contrast to an earlier report demonstrating that the USA300 isolate LAC did not form a biofilm in a microtiter plate assay [8]. Because we did not include LAC in our experiments, one possible explanation for this difference is strain-dependent variability even among USA300 isolates. However, this seems unlikely in that one of the USA300 isolates we included (UAMS-1782) is essentially indistinguishable from LAC as defined by both genotypic and phenotypic characteristics [39]. An alternative explanation is that we employed a different growth medium in our microtiter plate assay and, unlike the earlier study [8], coated the substrate with human plasma. With regard to the first, one possible explanation for our disparate results is that expression of *agr* was reduced in USA300 isolates under our growth conditions to the point that it had no impact on biofilm formation. To the extent that RNAIII levels remained relatively high in RN6390 even under these conditions, this is consistent with the observation that RN6390 was the only strain we examined in which mutation of *agr* had an impact on biofilm formation (see below).

**Figure 3:**
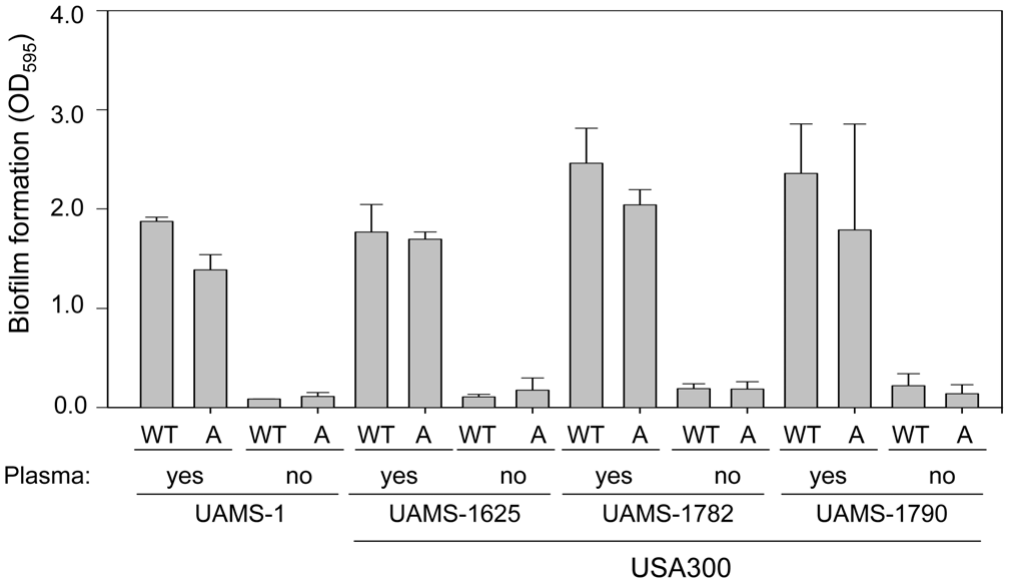
Impact of plasma coating on biofilm formation. Biofilm formation was assessed in UAMS-1 and each of three USA300 isolates (WT) and their isogenic *agr* (A) mutants using a static microtiter plate assay with and without plasma coating. Results are shown as the mean ± the standard deviation of 6 replicate samples. Statistical analysis confirmed a significant difference in all strains based on the presence vs. absence of plasma coating. No significant differences were observed between any wild-type strain and its isogenic *agr* mutant irrespective of whether plasma coating was employed.

Coating with plasma was also shown to play an important role. Specifically, we repeated our experiments using the same microtiter plate assay but without plasma coating and found that, as with the other clinical isolates we have examined [10], plasma coating enhanced biofilm formation in all USA300 isolates (Fig. 3). Thus, it seems likely that our use of supplemented TSB as a growth medium and plasma coating of the substrate both contributed to the ability of USA300 isolates to form a biofilm in our assay. While it is difficult to correlate *in vitro* results using any assay conditions with *in vivo* conditions, implanted medical devices are coated with plasma proteins *in vivo* [40]–[43]. Moreover, the results we have observed with our microtiter plate biofilm assay have in all cases been consistent with those obtained using a murine model of catheter-associated biofilm formation [13], [23], [25], [44]. This includes experiments done with the USA300 isolate UAMS-1625. Specifically, while we found that this strain had a reduced capacity to form a biofilm *in vivo* by comparison to UAMS-1, it was nevertheless capable of doing so to an extent that could be correlated with reduced antibiotic susceptibility [13]. We also confirmed the negative impact of mutating *sarA* on biofilm formation, and the positive impact of this on antibiotic susceptibility, in both UAMS-1 and UAMS-1625 under both *in vitro* and *in vivo* conditions [12], [13].

**Figure 4:**
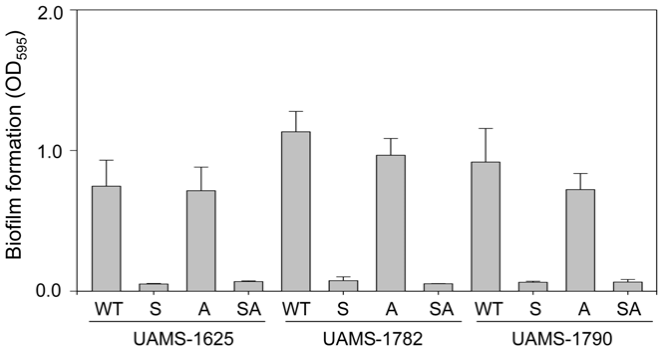
Impact of *sarA* and *agr* on biofilm formation in USA300 isolates. Biofilm formation was assessed in each of three USA300 isolates (WT) and their isogenic *sarA* (S), *agr* (A), and *sarA/agr* (SA) mutants using a static microtiter plate assay. Results are shown as the mean ± the standard deviation of 6 replicate samples. Statistical analysis confirmed a significant difference between each wild-type strain and its isogenic *sarA* and *sarA/agr* mutants but no difference between any wild-type strains and its *agr* mutant or between isogenic *sarA* and *sarA/agr* mutants.

We also found that mutation of *agr* did not enhance biofilm formation in any of the USA300 isolates irrespective of whether plasma coating was employed (Fig. 4) and this is consistent with the results of Lauderdale et al. [8] who found that mutation of *agr* in LAC had little impact on biofilm formation as assessed using flow cells. However, this certainly does not preclude an important role for *agr* in *S. aureus* biofilm formation, particularly given the relatively low levels of RNAIII production observed in USA300 isolates under our biofilm growth conditions. For instance, O'Neill et al. [45] demonstrated that mutation of *agr* enhanced biofilm formation in 5 of 13 methicillin-resistant clinical isolates even when the medium was supplemented with glucose to a degree that has been associated with reduced expression of *agr* [7]. Additionally, mutation of *sigB* in LAC limited biofilm formation even in flow cells, and concomitant mutation of *agr* reversed this effect [8].

### The impact of *sarA* on biofilm formation and its relationship to the production of extracellular proteases

Unlike *agr*, mutation of *sarA* was found to limit biofilm formation in all USA300 isolates irrespective of the functional status of *agr* (Fig. 4). O'Neill et al. [45] also found that mutation of *sarA* inhibited biofilm formation in all strains but suggested that this involved two different mechanisms, with the negative impact of mutating *sarA* in methicillin-sensitive strains being due primarily to the decreased expression of the *ica* operon leading to reduced production of the polysaccharide intracellular adhesin (PIA) while in methicillin-resistant strains the more important consideration was the impact of *sarA* on the production of surface-associated protein adhesins. We previously demonstrated that mutation of *sarA* in the methicillin-sensitive strain UAMS-1 does result in reduced PIA production, but an isogenic *ica* mutant retained the capacity to form a biofilm [27]. This demonstrates that decreased PIA production cannot account for the biofilm-deficient phenotype of a UAMS-1 *sarA* mutant. Rather, our results to date suggest that the more important consideration even in UAMS-1 is the increased production of extracellular proteases [23].

**Figure 5:**
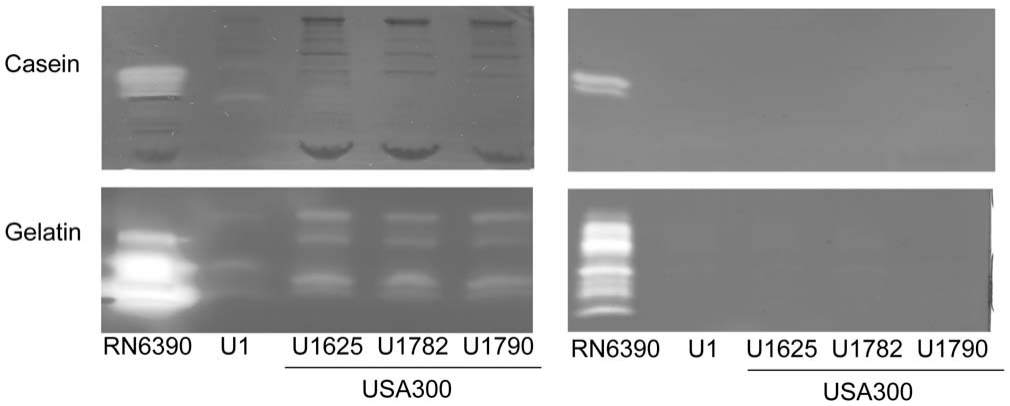
Production of extracellular proteases in USA300 isolates. Supernatants were harvested from overnight (15 hr) cultures grown in TSB (left) or biofilm medium (right) and standardized with respect to each other prior to zymographic analysis using both casein (top) and gelatin gels (bottom).

Production of these proteases has a negative impact on the presence of several surface-associated adhesins. These include the fibronectin-binding proteins (FnbA and FnbB) and protein A [24], [46], both of which contribute to biofilm formation in at least some clinical isolates of *S. aureus* [45], [47], [48]. Additionally, expression of *agr* both represses the production of these adhesins and induces the production of extracellular proteases [21], [49], either or both of which could contribute to the negative correlation between *agr* and biofilm formation. At the same time, transcription of the genes encoding extracellular proteases, including aureolysin (*aur*) and *sspA*, is directly repressed by both SarA and Rot [22]. Moreover, the regulatory impact of *sarA*, *rot* and *agr* appears to be dependent on the relative concentrations of their products with respect to each other rather than the concentration of any individual product alone [50]. This suggests that USA300 isolates may not produce extracellular proteases at the levels that might be expected based on their relatively high overall levels of *agr* expression, and we found that this was in fact the case. Specifically, by comparison to RN6390, all three USA300 isolates produced reduced amounts of all extracellular proteases that could be detected using either casein or gelatin zymography (Fig. 5). In fact, USA300 protease levels were more comparable to UAMS-1 than RN6390 despite the relatively low levels of RNAIII production in UAMS-1. As was observed in assays examining the production of RNAIII (Fig. 1), this was true whether protease production was assessed using supernatants from cultures grown in TSB or in biofilm medium (Fig. 5).

**Figure 6:**
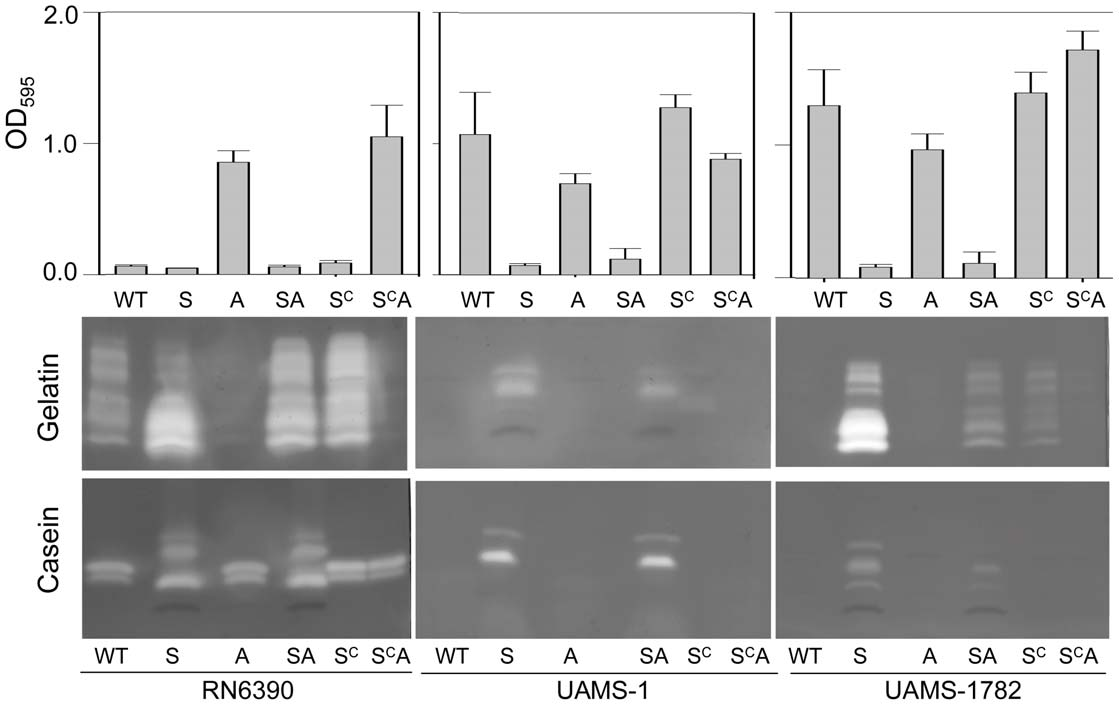
Relationships between *sarA, agr*, protease production and biofilm formation. Biofilm formation and the production of extracellular proteases was assessed in the indicated wild-type (WT) strains and their isogenic *sarA* (S), *agr* (A), and *sarA/agr* (SA) mutants with and without complementation with a functional copy of *sarA* (S^C^). Results for biofilm assay (top) are shown as the mean ± the standard deviation of 6 replicate samples. Statistical analysis confirmed a significant difference between the RN6390 *agr* and *sarA*-complemented RN6390 *sarA*/*agr* mutants and all other RN6390 derivative and between the *sarA* and *sarA/agr* mutants and their *sarA*-complemented derivatives in both UAMS-1 and UAMS-1782. No significant differences were observed the *sarA*-complemented derivatives of UAMS-1 or UAMS-1782 and their respective parent strains.

These results also suggest that the enhanced ability of USA300 isolates to form a biofilm by comparison to RN6390 might be a function of their decreased production of extracellular proteases. To further address this issue, we examined the relative impact of mutating *sarA* and *agr* on both biofilm formation and protease production, and in all cases we found a direct and inverse relationship between these two phenotypes. Specifically, the only strain derived from RN6390 that produced a biofilm in our microtiter plate assay was an *agr* mutant, which also exhibited decreased production of extracellular proteases (Fig. 6). Although mutation of *sarA* in RN6390 had relatively little impact of either of these phenotypes, concomitant mutation of *sarA* and *agr* in RN6390 reversed both phenotypes by comparison to the corresponding *agr* mutant (Fig. 6). This demonstrates that *sarA* is epistatic to *agr* even in RN6390 in the context of both protease production and biofilm formation. The same inverse relationships were also observed in UAMS-1 and all three of the USA300 isolates we examined, the difference being that the impact of *sarA* was evident in these strains irrespective of the functional status of *agr* (Fig. 6). These results confirm that the impact of *sarA* on protease production and biofilm formation is at least partially independent of *agr*. This was confirmed by demonstrating that the *sarA* defect was complemented in all strains, including RN6390, by introducing a functional, plasmid-borne copy of *sarA* even into a *sarA/agr* double mutant (Fig. 6).

**Figure 7:**
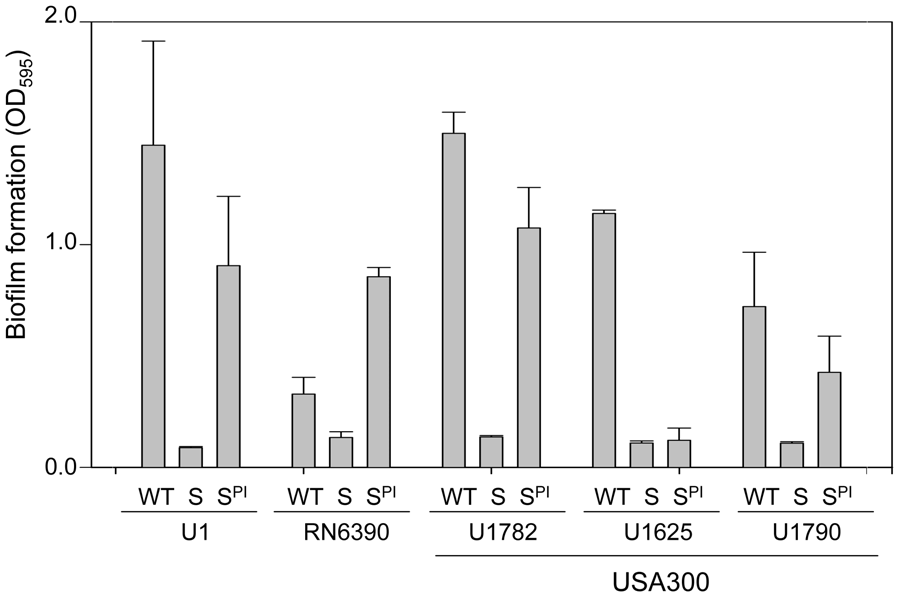
The impact of protease inhibitors on biofilm formation in *S. aureus sarA* mutants. Biofilm formation was assessed in each of the indicated strains (WT) and their isogenic *sarA* mutants with (S^PI^) and without (S) the inclusion of protease inhibitors. Results are shown as the mean ± the standard deviation of 6 replicate samples. Statistical analysis confirmed a significant difference between each wild-type strain and its *sarA* mutant and, with the exception of UAMS-1625, between the *sarA* mutants assayed in the presence or absence of protease inhibitors. In RN6390 and UAMS-1782, the difference between the wild-type strain and its isogenic *sarA* mutant assayed in the presence of protease inhibitors was also significant.

**Figure 8:**
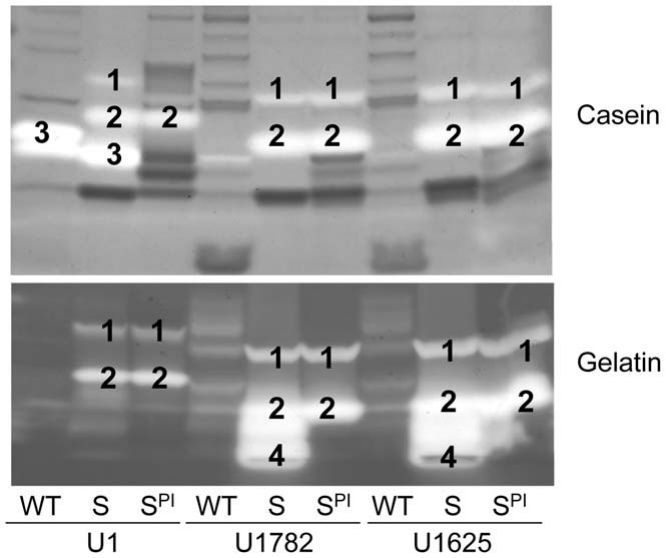
The impact of protease inhibitors on the activity of extracellular proteases. Supernatants from the wild-type strains (WT) and their isogenic *sarA* mutants with (S^PI^) and without (S) protease inhibitors were harvested from overnight cultures and standardized with respect to each other prior to zymographic analysis using both casein (top) and gelatin gels (bottom). Based on relative activity with casein vs. gelatin, molecular size, and known polymorphisms within the corresponding genes/proteins [49], the presumed identity of specific proteases are SspA (1), aureolysin (2), ScpA (3) and SspB (4). The identity of other proteases remains unknown.

While suggestive, the inverse relationship between biofilm formation and the production of extracellular proteases does not prove a cause-and-effect relationship. However, we subsequently demonstrated that a cocktail of three protease inhibitors that is capable of limiting the activity of multiple *S. aureus* extracellular proteases [23] enhanced biofilm formation in *sarA* mutants generated in UAMS-1, RN6390, and in two of the three USA300 isolates, the only exception being the USA300 isolate UAMS-1625 (Fig. 7). The overall levels of both RNAIII (Fig. 1) and extracellular proteases (Fig. 8) were comparable between UAMS-1625 and the other USA300 isolates, and the impact of mutating *sarA* on the production of extracellular proteases was comparable in all strains other than RN6390 (Fig. 8). This suggests that one possible explanation for the differential impact of protease inhibitors in UAMS-1625 is that this strain produces either a unique protease that was not inhibited by any component of the inhibitor cocktail or a common protease that is produced in elevated amounts by comparison to the other strains such that the inhibitor cocktail had a reduced effect in this strain. In this respect it is important to note that the inclusion of protease inhibitors did not fully restore biofilm formation (Fig. 7) or protease production (Fig. 8) in any of the *sarA* mutants. However, the relative impact of the inhibitor cocktail also appeared to be comparable among all USA300 isolates including UAMS-1625 (Fig. 8).

Interestingly, while the overall impact of the inhibitor cocktail was consistent in all strains we did observe a strain-dependent effect with respect to both the impact of mutating *sarA* and the relative efficacy of the protease inhibitor cocktail. Specifically, in UAMS-1, the impact of mutating *sarA* was most evident in the increased production of aureolysin and SspA, and the impact of the protease inhibitor cocktail was most evident in the reduced activity of SspA and ScpA (Fig. 8). In contrast, the impact of mutating *sarA* in USA300 isolates was most evident in the production of aureolysin, SspA and SspB, with the inhibitor cocktail having the greatest impact on the activity of SspB. The *spl*-encoded proteases have also been implicated in biofilm formation [8], but these were not detectable in either of our zymograms. These results are consistent with the hypothesis that multiple proteases contribute to the biofilm-deficient phenotype of *S. aureus sarA* mutants [7], [8], [23].

### The impact of *sarA* on production of the polysaccharide intercellular adhesin (PIA)

**Figure 9:**
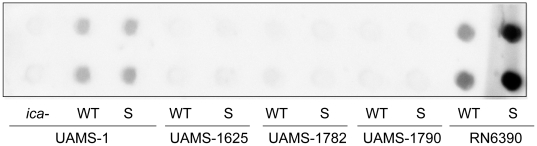
Role of *sarA* in production of the polysaccharide intracellular adhesin (PIA). PIA was isolated from each of the wild-type strains (WT) and their isogenic *sarA* mutants (S) and immunoblotted using anti-PIA serum. A UAMS-1 *ica* mutant was included as a negative control. Upper and lower rows are duplicate samples from each strain.

UAMS-1625 can also be distinguished from the other USA300 isolates included in this study by the absence of the arginine catabolite metabolic element (ACME) [38]. We are unaware of a correlation between ACME and biofilm formation, but there are reports describing the role of the *arc* operon itself in this context. Specifically expression of the *arc* operon was induced in UAMS-1 in a biofilm by comparison to both exponential and stationary-phase planktonic cultures [27]. Additionally, mutation of *arcD*, which encodes the arginine/ornithine antiporter of the arginine deiminase pathway, resulted in the reduced production of PIA in UAMS-1 [44]. However, as with the *ica* operon itself [27], this was not associated with a decreased capacity to form a biofilm. Nevertheless, the possibility that ACME contributes to biofilm formation by virtue of its impact on the production of PIA cannot be ruled out. Indeed, mutation of *sarA* results in reduced production of PIA [7], [11], and based on this one possible explanation for our results is that PIA plays a more important role in biofilm formation in UAMS-1625 than in the other USA300 isolates and that, in the absence of ACME, a UAMS-1625 *sarA* mutant cannot produce enough PIA to sustain biofilm formation irrespective of any other factor including extracellular proteases. To explore this possibility, we examined the relative levels of PIA produced by each strain using an anti-PIA immunoblot. These studies demonstrated that all of the USA300 isolates produced almost undetectable amounts of PIA irrespective of the presence of ACME or the functional status of *sarA* (Fig. 9). This not only suggests that the relative levels of PIA production do not account for the difference between UAMS-1625 and other USA300 isolates but also that PIA plays little role in USA300 biofilm formation. This is also consistent with previous reports demonstrating that mutation of *ica* had little effect on biofilm formation not only in LAC [8] but also in other methicillin-resistant *S. aureus* strains [45].

### The impact of *sarA* on nuclease production and its impact on biofilm formation

**Figure 10:**
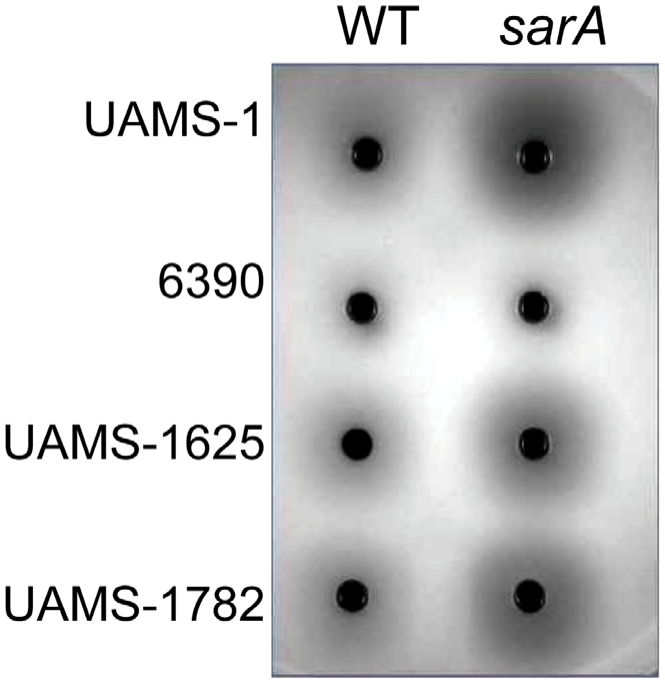
Impact of *sarA* on extracellular nucleases. The production of extracellular nucleases was assessed in the wild-type strains (WT) and their isogenic *sarA* mutants using DNase agar.

**Figure 11:**
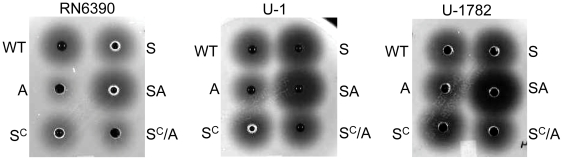
Epistatic relationship between *sarA* and *agr* in nuclease production. Biofilm formation and production of extracellular nucleases was assessed using DNase agar in the indicated wild-type (WT) strains and their isogenic *sarA* (S), *agr* (A), and *sarA/agr* (SA) mutants with (S^C^) and without complementation of the *sarA* defect.

Overall, our results are consistent with the hypothesis that the inability of *sarA* mutants to repress the production of extracellular proteases plays a predominant role with respect to their dominant-negative phenotype by comparison to *agr*. However, the inclusion of protease inhibitors did not fully restore biofilm formation in any of the *sarA* mutants (Fig. 7). One explanation for this partial effect is that the concentration of each inhibitor, which was chosen based on the highest concentration that did not inhibit growth [23], did not fully inhibit the activity of all extracellular proteases in UAMS-1 or any of the USA300 *sarA* mutants by comparison to their respective parent strains (Fig. 8). The alternative although not mutually exclusive explanation for the partial impact of protease inhibitors is that other factors also contribute to the biofilm-deficient phenotype of *sarA* mutants. Because extracellular DNA (eDNA) also contributes to biofilm formation in *S. aureus* [25], [26], one possibility in this regard is the production of extracellular nucleases. Indeed, we previously demonstrated that mutation of *sarA* in UAMS-1 resulted in increased production of nuclease and that mutation of *nuc* partially restored the ability of a UAMS-1 *sarA* mutant to form a biofilm [23]. In this report, we found that mutation of *sarA* also results in increased production of extracellular nucleases in USA300 isolates (Fig. 10). Moreover, the same epistatic relationships between *sarA* and *agr* that were observed in the context of biofilm formation and protease production were also observed in the context of nuclease production (Fig. 11). This demonstrates that the impact of *sarA* on nuclease production is also independent of the interaction between *sarA* and *agr* and that nuclease production and biofilm formation are also inversely correlated.

Concomitant mutation of *sarA* and *agr* resulted in increased nuclease activity in all strains even by comparison to the isogenic *sarA* mutant (Fig. 11). In this respect it is important to note that, while protease production remained elevated in *sarA/agr* mutants by comparison to the parent strains, it was reduced by comparison to the corresponding *sarA* mutants (Fig. 6). Thus, one possible explanation for these results is that the increased production of extracellular proteases limits the accumulation of extracellular nucleases and that the negative impact of mutating *agr* on protease production attenuates this effect. Additionally, UAMS-1 and all three isolates of the USA300 clonal lineage produced extracellular nucleases at levels that exceeded those observed in RN6390 (Figs. 10 and 11). Given its high level of protease production by comparison to clinical isolates, one possible explanation for this is that the increased production of extracellular proteases also limits nuclease activity in RN6390 irrespective of the functional status of *sarA*. However, the important point is that, because RN6390 was the only strain that did not form a biofilm even with plasma coating, these results are an exception to the inverse relationship between nuclease production and biofilm formation, and to the extent that no such exceptions were observed with respect to protease production, they are also consistent with the hypothesis that the production of extracellular proteases plays a predominant role by comparison to extracellular nucleases in *S. aureus* biofilm formation. Moreover, RN6390 was also found to produce more PIA than either UAMS-1 or the USA300 isolates (Fig. 9). Thus, of the three *sarA*-regulated components known to contribute to biofilm formation, RN6390 produces two (PIA and nuclease) at levels that would be expected to promote biofilm formation and one (protease) at levels that would be expected to limit biofilm formation. Taken together, these results provide further support for the hypothesis that the *sarA*-mediated repression of extracellular proteases plays a predominant role in *S. aureus* biofilm formation.

**Figure 12:**
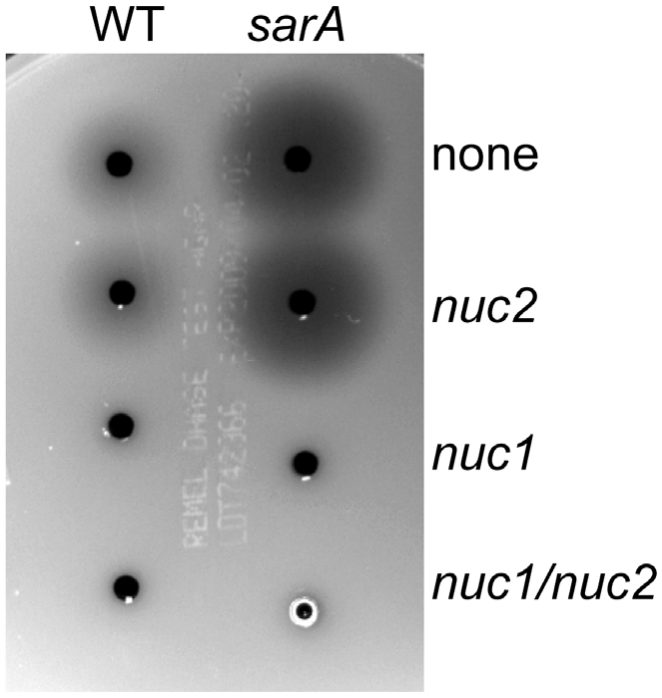
Activity of UAMS-1 nuclease genes. Nuclease activity was assessed using DNase agar in UAMS-1 (WT) and its *sarA* mutant and in derivatives of each of these strains carrying mutations in SA0746 (*nuc1*) and/or SA1160 (*nuc2*).

**Figure 13:**
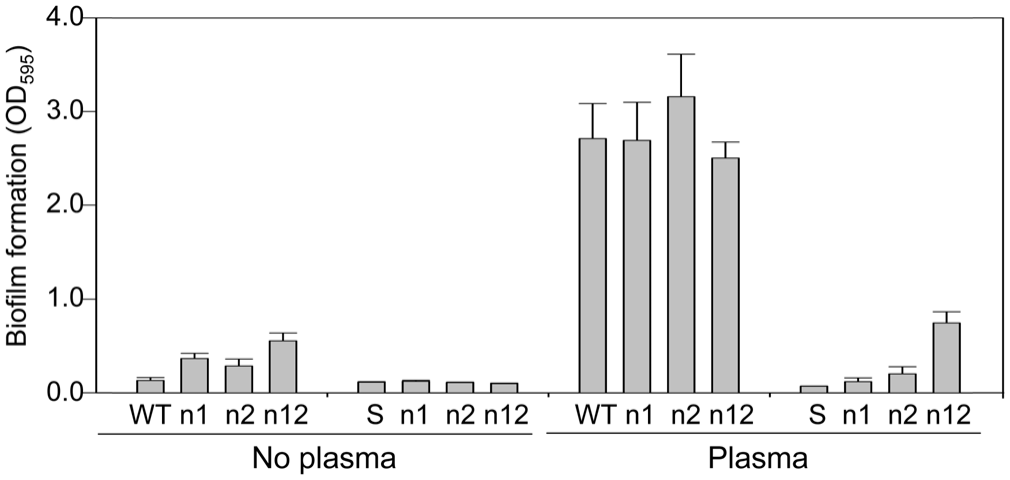
Impact of nuclease genes on biofilm formation. Biofilm formation was assessed in UAMS-1 (WT) and its *sarA* mutant (S) and in derivatives of each of these strains carrying mutations in *nuc1* (n1), *nuc2* (n2), or both *nuc1* and *nuc2* (n12) with and without plasma coating of the substrate. Results are shown as the mean ± the standard deviation of 6 replicate samples. Statistical analysis confirmed a significant difference between UAMS-1 (WT) and the isogenic *nuc1*, *nuc2* and *nuc1/nuc2* mutants in the absence of plasma coating and between the *sarA* mutant (S) and its isogenic *nuc1/nuc2* mutant in the presence of plasma coating.

At the same time, our previous results focusing on the impact of nuclease production on biofilm formation in UAMS-1 were done using a mutant generated in the *nuc* gene designated as open-reading frame SA0746 in the N315 genome, mutation of which was shown to abolish nuclease production even in a UAMS-1 *sarA* as assessed using DNase agar [23]. However, a recent report demonstrated the existence of two functional nuclease genes in *S. aureus*, one of which is encoded by SA0746 while the other is encoded by SA1160 [51]. These were designated *nuc1* and *nuc2* respectively. In these experiments we confirmed that mutation of *nuc1* in UAMS-1 essentially eliminated the production of extracellular nuclease even in a *sarA* mutant and that mutation of *nuc2* had no effect as assessed using DNase agar (Fig. 12). Even so, mutation of either or both of these genes was shown to enhance biofilm formation in UAMS-1 albeit to a limited degree and only in the absence of plasma coating (Fig. 13). This was also true in a UAMS-1 *sarA* mutant although in this case the effect was statistically significant only when both *nuc1* and *nuc2* were mutated and only when the assay was done with plasma coating. These results suggest that our failure to detect a nuclease-deficient phenotype in our *nuc2* mutant may have been due primarily to our use of a relatively insensitive assay. They also confirm that the increased production of nucleases contributes to biofilm formation in *S. aureus* at least under some circumstances and that the *agr*-independent, *sarA*-mediated repression of extracellular nuclease production may also be important in this regard.

### Summary

**Figure 14:**
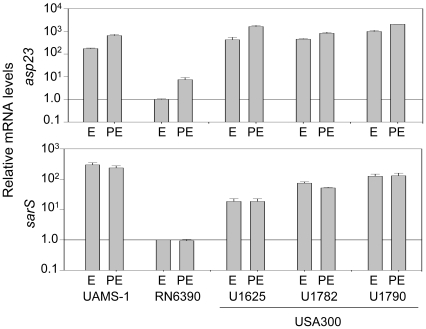
Expression of *asp23* and *sarS* in clinical isolates. RNA was isolated from each of the indicated strains during the exponential (E) and post-exponential (PE) growth phases and the amounts of the *asp23* and *sarS* transcripts determined by qRT-PCR. Values obtained with RN6390 exponential phase cultures were set to 1.0 with the results observed with other strains shown relative to this value. Results are shown as the mean ± the standard deviation of duplicate samples. Statistical analysis confirmed a significant difference between RN6390 and each of the other strains in the context of both *asp23* and *sarS* during both the exponential and post-exponential growth phases.

**Figure 15:**
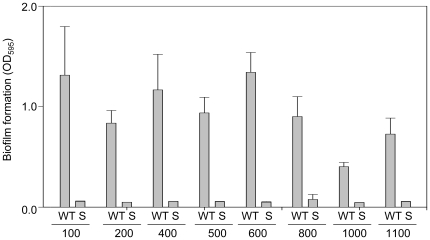
Impact of *sarA* on biofilm formation in isolates of other USA clonal lineages. Biofilm formation was assessed in wild-type strains (WT) from each of eight USA clonal lineages (numerical designations refer to USA clonal lineage) and their corresponding *sarA* mutants (S). Results are shown as the mean ± the standard deviation of 6 replicate samples. Statistical analysis confirmed a significant difference between each wild-type strain and its isogenic *sarA* mutant.

Taken together, the results discussed above demonstrate that *sarA* is epistatic to *agr* in the context of biofilm formation and that this is true irrespective of the level of *agr* expression or the overall genetic relatedness among clinical isolates. The results also suggest that the primary regulatory role of *sarA* in this context is to repress the production of extracellular enzymes including proteases and nucleases. They also suggest that in the specific context of these phenotypes the regulatory events observed in RN6390 reflect an imbalance by comparison to clinical isolates including those of the USA300 clonal lineage. This imbalance is defined both by the absolute level of *agr* expression and the inability of RN6390 to modulate expression of *agr*, and consequently the predominance of *agr* relative to *sarA*, and this is directly reflected in the increased production of extracellular proteases in RN6390. This presumably reflects the fact that all 8325-4 strains, including RN6390, carry mutations in at least two genes (*rsbU* and *tcaR*) known to impact *S. aureus* regulatory circuits [22], [29]. This is consistent with the observation that restoration of *rsbU* in an 8325-4 strain (SH1000) resulted in reduced production of extracellular proteases [28]. To the extent that RN6390 was the only strain we examined in which mutation of *agr* resulted in an enhanced capacity to form a biofilm (Fig. 6), it is also consistent with the observation that the impact of mutating *agr* on biofilm formation in LAC was most evident in an *agr/sigB* mutant [8]. Other reports have suggested that the impact of the *tcaR* mutation on expression of *sarS* also has a profound impact on *S. aureus* regulatory circuits [22], [50]. As evidenced by expression levels of *asp23*, which is indicative of the functional status of *rsbU* (or, more precisely, *sigB*) and *sarS*, neither of these mutations is present in any of the clinical isolates examined in this report (Fig. 14). However, to the extent that RN6390 has these defects, the more important observation is that mutation of *sarA* resulted in a significantly reduced capacity to form a biofilm in all of the isolates we examined other than RN6390. Moreover, we extended our experiments to include other clonal lineages of *S. aureus*, and in every case mutation of *sarA* resulted in a reduced capacity to form a biofilm to a degree that was comparable to that observed in UAMS-1 and isolates of the USA300 clonal lineage (Fig. 15).

We recently demonstrated that mutation of *sarA* in UAMS-1 and the USA300 isolate UAMS-1625 can be correlated with increased antibiotic susceptibility both *in vitro* and *in vivo* [12], [13], and our results demonstrating a comparable effect in other clinical isolates suggest that this would be the case irrespective of strain identity. This is consistent with the hypothesis that inhibitors of *sarA*-mediated regulation would have broad therapeutic utility in the specific context of biofilm-associated staphylococcal infection. While such inhibitors would have neither bacteriostatic or bactericidal properties and thus would have limited therapeutic utility in and of themselves, many *S. aureus* infections are recalcitrant to antimicrobial therapy even in the absence of issues related to acquired resistance. This includes orthopaedic and catheter-associated infections, the resolution of which often requires surgical intervention to debride infected tissues and/or removal of the infected device [42], [43], [52], [53]. Based on this, such inhibitors would potentially be a viable alternative for the development of adjunct therapies that could be used to significantly enhance the efficacy of more conventional antimicrobial agents in the specific context of *S. aureus* biofilm-associated infection. The results we report demonstrate that the impact of *sarA* on biofilm formation is epistatic to *agr*, and this implies that, irrespective of the specific mechanism involved, such inhibitors would maintain their efficacy even in the context of the accumulation of *agr* mutants that occurs not only within biofilms [6] but also *in vivo* during the course of antimicrobial therapy [15].

## Materials and Methods

### Bacterial strains and growth conditions

The *S. aureus* strains used in this study are listed in Table 1. Generation of *agr*, *sarA* and *agr/sarA* mutants in each of the targeted strains was done by Φ11-mediated transduction of the *agr::tet*, *sarA::tet* or *sarA::kan* mutations from existing strains [24]. Complementation of the *sarA* mutation was done as previously described [24]. Mutagenesis of the SA0746-encoded nuclease gene (designated here as *nuc1*) in UAMS-1 and its isogenic *sarA* mutant was done using pKOR1 as previously described [7]. The UAMS-1 SA1160 *nuc* mutant (designated here as *nuc2*) was generated by allele replacement mutagenesis. Specifically, 498 and 469 bp fragments were independently amplified from the upstream and downstream regions of the targeted gene using the primers shown in Table 1. These fragments were cloned into pGD647 [54] on either side of the *erm* gene using the EcoRI and KpnI restriction sites for the upstream fragment and XbaI and PstI restriction sites for the downstream fragment. The resulting *nuc2*-erm-*nuc2* cassette, which contains all of the SA1160 gene except the 115 bp region that was replaced with the 1.3 kb *erm* gene, was then excised by EcoRI/PstI digestion and subcloned into pCL52.2 [55]. After passage through RN4220 and electroporation into UAMS-1, allele replacement was accomplished by growth and 45°C followed by repetitive culture without antibiotic selection at 30°C. Colonies were plated on tryptic soy agar (TSA) without antibiotic and then patched to TSA containing erythromycin or tetracycline. Colonies resistant to erythromycin and sensitive to tetracycline were then screened by PCR to confirm allele replacement using the 5′ primer from the upstream PCR and the 3′ primer from the downstream PCR. The resulting *nuc2* mutation was subsequently introduced into the isogenic *nuc1*, *sarA* and *sarA/nuc1* mutants by phage-mediated transduction [7].

**Table 1:** Bacterial strains used in this study.

Strain	Description	Reference
UAMS-1	MSSA, osteomyelitis isolate	[56]
UAMS-128	RN6390 (8325-4)	[57]
UAMS-155	UAMS-1agr::tetM	[24]
UAMS-240	UAMS-128, sarA::tetK	[24]
UAMS-929	UAMS-1, sarA::kan	[24]
UAMS-930	UAMS-929, agr::tetM	[24]
UAMS-959	UAMS-983, agr::tetM	[24]
UAMS-969	UAMS-929, (pLI50::sarA)	[24]
UAMS-970	UAMS-930, (pLI50::sarA)	[24]
UAMS-979	UAMS-983, (pLI50::sarA)	[24]
UAMS-980	UAMS-959, (pLI50::sarA)	[24]
UAMS-982	UAMS-128, agr::tetM	[24]
UAMS-983	UAMS-128, sarA::kan	[24]
UAMS-1039	USA400 isolate (MW2)	NRS123^1^
UAMS-1454	UAMS-1 nuc2::erm	This study
UAMS-1471	UAMS-1 nuc1	[23]
UAMS-1477	UAMS-929 nuc1	[23]
UAMS-1478	UAMS-1 nuc1, nuc2::erm	This study
UAMS-1484	UAMS-929 nuc2	This study
UAMS-1485	UAMS-929, nuc1, nuc2::erm	This study
UAMS-1625	USA300 isolate	[38]
UAMS-1653	UAMS-1625, sarA::tetK	[12]
UAMS-1660	UAMS-1625, agr::tetM	This study
UAMS-1782	USA300 isolate FPR3757	NRS482
UAMS-1790	USA300 isolate	[37]
UAMS-1796	UAMS-1790, sarA::tetK	This study
UAMS-1804	UAMS-1782, sarA::kan	This study
UAMS-1819	UAMS-1782, agr::tetM	This study
UAMS-1820	UAMS-1790, agr::tetM	This study
UAMS-1836	UAMS-1660, sarA::tetK	This study
UAMS-1837	UAMS-1819, sarA::kan	This study
UAMS-1838	UAMS-1820, sarA::tetK	This study
UAMS-1893	USA100 isolate	NRS642
UAMS-1894	USA200 isolate	NRS651
UAMS-1895	USA500 isolate	NRS685
UAMS-1896	USA600 isolate	NRS648
UAMS-1898	USA800 isolate	NRS653
UAMS-1899	USA1000 isolate	NRS676
UAMS-1900	USA1100 isolate	NRS484
UAMS-1901	UAMS-1804, (pLI50::sarA)	This study
UAMS-1904	UAMS-1837, (pLI50::sarA)	This study
UAMS-1930	UAMS-1899, sarA::kan	This study
UAMS-1931	UAMS-1900, sarA::kan	This study
UAMS-1938	UAMS-1039, sarA::kan	This study
UAMS-1941	UAMS-1893, sarA::tetK	This study
UAMS-1942	UAMS-1895, sarA::tetK	This study
UAMS-1943	UAMS-1896, sarA::tetK	This study
UAMS-1944	UAMS-1898, sarA::tetK	This study
UAMS-1945	UAMS-1894, sarA::tetK	This study

1 NRS isolates were obtained from the repository at the Network for Antimicrobial Resistance in Staphylococcus aureus (NARSA), Eurofins Medinet, Inc., Chantilly, VA 20151.

All strains were maintained as stock cultures at −80°C in tryptic soy broth (TSB) containing 25% (v/v) glycerol. For each experiment, each strain was retrieved from cold storage by plating on tryptic soy agar (TSA) with appropriate antibiotic selection. Antibiotics were used at the following concentrations: erythromycin (Erm; 10 µg per ml), tetracycline (Tet; 5 µg per ml), kanamycin (Kan; 50 µg per ml), neomycin (Neo; 50 µg per ml), and chloramphenicol (Cm; 10 µg per ml). Kanamycin and neomycin were always used together to avoid the spontaneous generation of resistant strains. To ensure that the results of all phenotypic assays were consistent, unless otherwise noted all assays were done using cultures grown in TSB supplemented with 0.5% glucose and 3.0% sodium chloride (biofilm medium) without antibiotic selection as previously described [7], [10], [27]. Culture conditions in all cases were 37°C with constant aeration and a medium-to-flask volume ratio of ≤0.50. In experiments evaluating the impact of growth phase, the exponential and post-exponential growth phases were defined based on optical densities (OD) of 1.0 and 3.0 respectively. Stationary phase samples were defined by overnight (24 hr) growth.

### Transcriptional analysis

To assess levels of *asp23*, *sarS* and RNAIII expression, total bacterial RNA was isolated using the Qiagen RNeasy Mini Kit as previously described [23]. Quantitative, real-time RT-PCR (qRT-PCR) was then performed using RNAIII-specific primers and a corresponding TaqMan probe (Table 2). Results were calibrated by comparison to the results obtained with the same RNA samples using primers and a TaqMan probe corresponding to a 16S ribosomal RNA gene (Table 2). Results are reported as relative units by comparison to the results observed with the lowest sample in any given experiment, with the latter being set to a value of 1.0.

**Table 2:** Oligonucleotide primers and probes used in this study.

Gene	Primer or probe	OligonucleotideSequence^1^
RNAIII	probe	56-FAM\TGTGCCATTGAAATCACTCCTTCC\3BHQ1
	forward	ATGGAAAATAGTTGATGAGTTG
	reverse	CTAAGTCACCGATTGTTGAA
*sarS*	probe	56-FAM\TGGTCTTGCTGCGCGTCATCC\3BHQ1
	forward	AAATACCCTCAAACTGTTAGAGC
	reverse	TCACTTGAGCTAATAATTGTTCAG
*asp23*	probe	56-FAM\ACAAGCATACGACAATCAAACTGGT\3BHQ1
	forward	CAACAACTTCATCAGAGAATG
	reverse	ATGACTGTAGATAACAATAAAGC
16S	probe	56-FAM/AGCGCAACCCTTAAGCTTAGTTGCCA/3IABlkFQ
	forward	TGAGATGTTGGGTTAAGTCCCGCA
	reverse	CGGTTTCGCTGCCCTTTGTATTGT
nuc2	upstream forward	GAGACTTGGAAAGTGAGTCAA
	upstream reverse	TGAAAGGACCCGTATGATT
	downstream forward	AGTTAGGCTTATAGGGGTTGA
	downstream reverse	TAAAATCAAAGGCATAATAATCCA

1 All primer sequences are written 5′ to 3′.

### Assessment of biofilm formation

Biofilm formation was assessed *in vitro* using a static, microtiter plate biofilm assay as previously described [7], [10]. Unless otherwise indicated, the microtiter plate substrate was first coated overnight with 20% human plasma.

### Production of extracellular proteases

Protease activity was assessed by zymography using 4–16% Zymogram (Blue Casein) Gels and 10% Zymogram (Gelatin) Gels (Invitrogen, Carlsbad, CA). In both cases, supernatants were harvested from overnight (15 hr) cultures and normalized with respect to each other prior to filter sterilization. Sterile supernatants were then concentrated 15-fold using Centricon YM-3 filter units (Millipore, Bedford, MA) before loading equivalent samples using a buffer containing DTT but not β-mercaptoethanol. After electrophoresis, gels were first incubated for 30 min at room temperature (RT) in renaturing buffer (2.5% TritonX-100) and then overnight at 37°C in developing buffer (0.2 M Tris, 5 mM CaCl_2_, 1 mM DTT). To visualize protease bands, gels were then stained with SimplyBlue SafeStain (Invitrogen, Carlsbad, CA) at RT for 2 hrs before destaining overnight in distilled water. Assays employing the E-64, 1-10-phenanthroline (Fisher Scientific, St. Louis, MO) and dichloroisocoumarin (DIC) (Sigma Chemical Co., St. Louis, MO) protease inhibitors were done as previously described using the highest concentration of each inhibitor that did not limit growth (4).

### Production of extracellular nucleases

Nuclease production was assessed using D'NASE Test Agar (REMEL, Lenexa, KS). Briefly, supernatants were harvested from overnight (15 hr) cultures and normalized with respect to each other prior to filter sterilization. Sterile supernatants were then concentrated 15-fold Centricon YM-3 filter units (Millipore, Bedford, MA). 15 µl aliquots were then placed into wells cut into the D'NASE test agar. Plates were incubated overnight at 37°C. Nuclease activity was then assessed by overlaying the agar with 1N HCl to precipitate undigested DNA and define the zone of clearance around the supernatant harvested from each strain.

### Production of the polysaccharide intercellular adhesin (PIA)

To assess the production of PIA, overnight cultures were normalized with respect to each other before harvesting cells by centrifugation. Cells were resuspended in 50 µl of 0.5 M EDTA (pH 8.0) and boiled for 5 min. Cellular debris was removed by centrifugation before incubating the supernatant with proteinase K (20 mg/ml) at 37°C for 60 minutes. After the addition of 10 µl of Tris-buffered saline (20 mM Tris-HCl, 150 mM NaCl [pH 7.4]), each extract was spotted onto a nitrocellulose membrane using a BIO-Dot microfiltration apparatus (Bio-Rad Laboratories, Inc., Hercules, CA). After drying, the presence and amount of PIA was assessed using anti-PIA antiserum and the WesternBreeze chemiluminescence immunodetection kit (Invitrogen Corp., Carlsbad, CA).

### Statistical analysis

Statistical analysis of results comparing wild-type strains was done using the Students t-test. Statistical analysis of results comparing different strains with their isogenic *sarA* and *agr* mutants and their *sarA*-complemented derivatives was done by ANOVA based on all pairwise comparisons. In both cases *p* values <0.05 were considered significant.
